# Bisphenol Chemicals in Surface Soil from E-Waste Dismantling Facilities and the Surrounding Areas: Spatial Distribution and Health Risk

**DOI:** 10.3390/toxics12060379

**Published:** 2024-05-23

**Authors:** Lei Zhao, Fengli Zhou, Shuyue Wang, Yan Yang, Haojia Chen, Xufang Ma, Xiaotu Liu

**Affiliations:** 1 Guangdong Key Laboratory of Environmental Pollution and Health, College of Environment and Climate, Jinan University, Guangzhou 510632, China; lei_zhao1998@163.com (L.Z.); zhoufengli@stu2020.jnu.edu.cn (F.Z.); wangshuyue@stu2022.jnu.edu.cn (S.W.); xufang_ma2002@163.com (X.M.); 2 School of Environmental Science and Engineering, Institute of Environmental Health and Pollution Control, Guangdong University of Technology, Guangzhou 510006, China; yangyan1209@gdut.edu.cn (Y.Y.); chenhaojia_gdut@163.com (H.C.); 3 Synergy Innovation Institute of Guangdong University of Technology, Shantou 515041, China; 4 Chemistry and Chemical Engineering Guangdong Laboratory, Shantou 515041, China

**Keywords:** bisphenol chemicals, e-waste dismantling facilities, surface soil, daily intake

## Abstract

Electronic waste (e-waste) dismantling facilities are well-known bisphenol chemical (BP) sources. In this study, non-targeted screening combined with targeted analysis of BPs in surface soil from e-waste dismantling facilities and their surroundings revealed their presence, distribution, and exposure risk. A total of 14 BPs were identified including bisphenol A (BPA) and its novel structural analogs and halogenated BPs. The total concentrations of BPs ranged from 963 to 47,160 ng/g (median: 6970 ng/g) in e-waste soil, higher than those measured in surface soil from surrounding areas, i.e., 10–7750 ng/g (median 197 ng/g). BPA, tetrabromobisphenol A (TBBPA), and bisphenol F (BPF) were the dominant ones from the two areas. Concentrations of TBBPA and its debromination product from the surrounding area significantly decreased with increasing distances from the e-waste dismantling facilities. Estimation of daily intake via oral ingestion of soil suggests that current contamination scenarios are unlikely to pose health risks for e-waste dismantling workers and adults and toddlers living in the surrounding areas, with their intakes generally well below the tolerable daily intakes proposed for several BPs. However, the BPA intakes of workers exceeded the more strict tolerable daily intake for BPA established recently, which merits continuous environmental surveillance.

## 1. Introduction

Bisphenol chemicals (BPs) refer generically to compounds that possess two phenol groups in their structure [1]. Among the BPs documented for use in industrial applications, bisphenol A (BPA) stands out as one of the most recognized compounds globally [2]. BPA is widely used in the synthesis of epoxy resin and polycarbonate plastic as an organic synthetic additive, as well as in plastic items including toys and drinking containers [3]. Increasing demand for plastic materials has resulted in ubiquitous environmental distributions of BPA [4]. Increasing evidence suggests that BPA could potentially have harmful effects on humans, ranging from endocrine disruption and developmental toxicity to carcinogenicity, obesity, and reproductive toxicity [5,6,7]. Therefore, the use of BPA is restricted in many countries due to concerns about health and ecological risks from exposure [1,7].

The regulation of BPA has led to the use of an increasing number of alternatives. Over 200 bisphenol alternatives with structural similarities to BPA have been documented [8]. Due to structural similarities, some of those alternatives, such as bisphenol F (BPF) and bisphenol S (BPS), have also been found to have endocrine-disrupting effects [9]. This sparked concerns regarding the possible environmental exposures and health risks of the diverse range of these alternatives. In addition, halogenated BPs, such as tetrabromobisphenol A (TBBPA), are also an important class of BPs and have raised concerns recently [10]. Although there have been studies focusing on the environmental presence and human exposure to bisphenols other than BPA [1], due to the complexity of BPs, there might be a large number of unknown BPs that have not yet been identified.

Electronic waste (e-waste) disassembly is a significant source of many additive-based substances [11]. E-waste contains up to 30% plastic materials and epoxy resins (by weight), which are the main uses of BPs [12]. The e-waste dismantling process might result in the uncontrolled release of large quantities of these substances into the environment [13]. Previous studies have found high levels of BPA and its analog alternatives and halogenated derivatives of BPA in various environmental matrices around e-waste dismantling areas [14,15,16]. However, no study has systematically screened BPs in e-waste dismantling areas, and information remains limited other than BPA and TBBPA. In addition, as a significant source, the influences of e-waste disassembly activities on the concentrations of BPs in the surrounding environment have rarely been studied.

Recently, we have developed a non-targeted screening strategy D-ISF for the identification of BPs based on dansyl chloride (DnsCl) derivatization and positive electrospray ionization high-resolution mass spectrometry (HRMS) in-source fragmentation [17]. The strategy largely enhanced the detection sensitivities and accuracy by introducing easily ionizable functional groups to BPs and generating characteristic fragments. In light of D-ISF, this study aims to (1) screen BPs in surface soil samples collected from two typical e-waste dismantling sites in South China in a non-targeted manner; (2) characterize the concentrations and spatial distribution of all identified BPs in surface soil from e-waste dismantling facilities and the surrounding areas; and (3) estimate the exposure risks of BPs for occupation workers and residents living around the dismantling sites.

## 2. Materials and Methods

### 2.1. Sample Collection

Two typical large-scale e-waste dismantling parks in Qingyuan and Shantou, South China, were selected, and a total of 24 surface soil samples were gathered from various locations within the two industrial parks, including e-waste storage areas, dismantling operation zones, internal roadways, etc. Surface soil samples from the surroundings (including residential areas, commercial areas, schools, etc.) within a radius of 8 km of the e-waste dismantling park of Shantou were also collected (n = 34). Detailed information on sampling sites is listed in Table S1 and Figure S1 of Supplementary Materials. At each sampling site, soil was collected using precleaned brushes and wrapped with clean aluminum foil on consistently sunny days in 2021. Soil samples were sieved by passage through a 125 μm stainless sieve (Hogentogler & Co., Inc., Columbia, MD, USA) to remove some large stones and kept at −20 °C until analysis at the analytical laboratory.

### 2.2. Chemicals and Sample Preparation

All the standards and reagents in this study were purchased commercially, of which the purity of the standards was above 95% and the reagents were of high-performance liquid chromatography purity (details are provided in the Supplementary Materials). A previously established method for deriving dansyl chloride was applied for sample preparation. Approximately 50 mg of the soil sample was weighted and then spiked with 10 µL surrogate standards and vortexed (L600, Hunan Xiangyi Laboratory Instrument Development Co., Ltd., Changsha, China). Then, 3 mL of acetonitrile (ACN) was added for extraction. After sonication for 15 min (EFAA-DC24, ANPEL Laboratory Technologies Shanghai Inc., Shanghai, China), the extract was centrifuged at 2500 rpm for 5 min. After being repeated twice, the combined extract was concentrated to about 100 µL. The concentrated extract was then derivatized with DnsCl using the procedures described previously [17].

### 2.3. Instrumental Analysis

The screening of derivatized BPs in pooled e-waste soil using the D-ISF strategy was carried out on an ultra-performance liquid chromatography (UPLC)-HRMS device (Vanquish Flex, Thermo Fisher Scientific, Pleasanton, CA, USA) equipped with an Orbitrap Exploris 240 (Thermo Fisher Scientific, CA, USA). Detailed information was provided in our previous study [17]. The determination of identified derivatized BPs was performed on an Agilent 1290 Infinity LC coupled to a 6470 triple quadrupole MS equipped with Jet Stream Technology Ion Source electrospray ionization (AJS-ESI) (Agilent Technologies, Santa Clara, CA, USA). The conditions in the LC section, including column and mobile phases as well as the gradients, were as the same as those in the UPLC-Orbitrap Exploris 240 MS [17]. Derivatized BPs were detected under positive AJS-ESI in the multiple reaction monitoring (MRM) mode. The settings of the AJS-ESI source were as follows: the nozzle and capillary voltage were 1500 and 3000 V, respectively, and the sheath gas flow rate and temperature were 11 L/min and 250 °C, respectively.

### 2.4. Quality Assurance and Quality Control (QA/QC)

The performance of the DnSCl derivatization methods was evaluated with satisfactory results. For example, the derivatization efficiencies of target BPs ranged from 91.8% to 99.8% in solvents [17]. In this study, BPA, BPF, BPS, bisphenol E (BPE), bisphenol B (BPB), bisphenol G (BPG), and bisphenol Z (BPZ) were used as target chemicals for QA/QC. The matrix effects of derivatized BPs in soil samples ranged from 65.4 ± 5.6% to 114 ± 3.8%. The method recoveries were measured by spiking 20 ng of each of the target BPs into pooled soil samples collected from the university campus and processed in five replicates. The recoveries of the spiked BPs ranged from 65.6 ± 3.1% to 85.6 ± 3.6% after subtracting the original concentrations measured in soil. Two blanks were processed along with every ten soil samples. No target bisphenol compounds were detected in the procedural blanks. The calibration curves for the derivatized bisphenol standards exhibited linear regression coefficients > 0.99 with a range of 10 to 200 ng/mL. Additionally, the limits of detection (LODs), defined as a response 3 times the standard deviation of the noise, and the limits of quantification (LOQs), defined as a response 10 times the standard deviation, are provided in Table S2.

### 2.5. Exposure Assessment

The estimated daily intake of BPs (*EDI*, ng/kg BW/day) via oral ingestion of soil was calculated using the following equation [18]:

$$EDI=\frac{C\times DIR\times EF}{BW}$$

where *C* (ng/g) is the concentration of BPs in the soil, *DIR* represents the ingestion rate (g/day), *EF* indicates the exposure fraction (unitless, hours spent outdoors over a day), and *BW* stands for body weight (kg). The values of these parameters used for the assessment of e-waste dismantling workers and residents (adults and toddlers) living in the surrounding area are summarized in Table S3.

The hazard quotient (*HQ*) approach was used to estimate the potential health risk of exposure to BPs via oral ingestion:

$$HQ=\frac{EDI}{tolerable\;daily\;intakes}$$

A value of 4 μg/kg bw/day for the tolerable daily intake (TDI) of BPA was used in the study [19]. The TDI values for BPS, BPF, and TBBPA were 4.4, 3.5, and 1000 μg/kg bw/day, respectively [20,21], while TDIs for other BPs are not available.

### 2.6. Data Analysis

For BPs with a detection frequency exceeding 60%, if their measured values are below the LOQs, a value of LOQ/√2 will be assigned for statistical analysis. We used Spearman’s correlation analysis (two-tailed) to determine the associations between the concentrations of individual BPs and between the concentrations and the distances from the e-waste dismantling facilities to the sampling sites (PASW Statistics 18.0, IBM Inc., Armonk, NY, USA). Additionally, for the comparisons of concentrations of BPs between soil samples from the dismantling area and the surrounding area, the Mann–Whitney U test was utilized.

## 3. Results and Discussion

### 3.1. Screening of Bisphenol Chemicals in E-Waste Soil

A total of 24 potential BP candidates were screened out of the pooled samples of e-waste soil. After manually checking and confirming MS/MS spectra, 14 BPs were identified (Table S2). Among these, BPA, BPE, BPF, BPB, BPG, BPS, BPZ, bisphenol TMC (BP-TMC), 3-monobromobisphenol A (monoBBPA), and TBBPA were confirmed and quantified using reference standards. The other four BPs whose commercial standards are not available, including monochlorobisphenol A (monoClBPA), dibromobisphenol A (DiBBPA), tetramethyl bisphenol A (TMBPA), and 3,3’,5-Tribromobisphenol A (TriBBPA), were identified as follows: their measured MS spectrum matched with the theoretical isotopologue distributions and some characteristic fragments were observed in their MS/MS spectrum (Figures S2–S5). The identified BPs were analyzed in individual samples using MRM mode in LC-MS. A pseudo-MRM method was developed for the four BPs without standards and they were semi-quantified using standards of other compounds (Table S4) [22].

### 3.2. Concentrations and Profiles of Bisphenol Chemicals in Surface Soil

The concentrations and detection frequencies (DFs) of BPs in soil samples from e-waste dismantling facilities and the surrounding areas are shown in Table 1. A total of nine BPs had DFs of 100% in e-waste samples, including BPA, BPF, BPB, TMBPA, TBBPA, monoClBPA, monoBBPA, DiBBPA, and TriBBPA, while only BPA and BPF were detected in all samples from surrounding areas. The total concentrations of all identified BPs (referred to as ΣBPs) ranged from 962 to 47,165 ng/g with a median of 6968 ng/g in e-waste soil. These were significantly elevated compared to those detected in soil samples from the surroundings, i.e., 10.3–7751 ng/g (median: 197 ng/g) (*p* < 0.001). The data suggest that BPs are widely present in the e-waste dismantling environment and that e-waste dismantling activities may be an important source of bisphenols.

BPA dominated over other BPs present in both e-waste soil and surrounding area surface soil, accounting for 53 ± 9.0% and 54 ± 10.5% of ΣBPs, respectively (Figure S6), indicating that BPA remains the most widely used bisphenol in China. The concentrations of BPA in e-waste soil ranged from 531 to 167,00 ng/g, with a median of 3350 ng/g, which were significantly higher than those observed in surrounding area samples, i.e., a median of 43 ng/g (*p* < 0.001). Our BPA concentrations were also much higher compared to previous studies which reported the concentrations in dust or soil from e-waste dismantling areas. For example, the mean concentration was 4.59 ng/g in soil samples from Taizhou [16], 49 ng/g from Xian [23], and 140 ng/g from India [14]. However, our concentration was comparable to those in indoor dust, also collected from South China (mean, 19,200 ng/g) [15]. This difference may be related to the type of waste handled by the e-waste dismantling workshop. In comparison with studies conducted in non-electronic-waste dismantling areas, the detection frequencies and concentrations in our studies were much higher. For example, our concentrations in the surrounding area soil far exceed those in soil samples collected from 21 provinces in China [24]. The notably high BPA concentrations in e-waste soil might be attributed to the burning of BPA-containing computer-printed circuit boards [25].

Seven structural analogs of BPA were widely detected, including BPF, BPE, BPB, BPS, BPZ, BP-TMC, and BPG. Among these, BPF was the dominant one with the highest concentrations (median, 920 ng/g and 12.3 ng/g for e-waste and surrounding area, respectively). In contrast, BPS was only detected in 75% of e-waste samples and 9% of surrounding area samples. The concentrations of BPS in e-waste soil (median, 9.86 ng/g) were much lower than those in household dust collected from Guangzhou (median, 320 ng/g) [26]. The low concentrations and detection rates of BPS in the e-waste dismantling areas were in line with previous studies [15]. A biomonitoring study showed that people living near e-waste recycling facilities contained significantly higher levels of BPF in their urine than those living in rural areas, but there was no such trend for BPS [13]. The high concentration of BPF in e-waste soil could be ascribed to the utilization of BPF as the primary alternative to BPA for epoxy resin and plastic synthesis [1]. By contrast, BPS is used in a wider range of consumer products, including paper and personal care products [27]. In addition to BPF and BPS, BP-TMC, BPG, BPE, BPB, and BPZ were rarely detected in previous studies. Their widespread detection suggests that they are also widely used in products related to e-waste.

As a structurally similar substance to BPA, TMBPA is primarily used as an intermediate in synthesizing polycarbonate resin [28]. A series of studies have compared the toxicity of BPA and TMBPA. TMBPA has been reported to be more potent than BPA in inducing lipid deposition in HepG2 cells [29], and similar to BPA in inducing estrogenic activity in human breast cancer cells [28]. However, to date, no study has investigated the environmental occurrence of TMBPA. Our data showed that TMBPA was detected in all e-waste samples with semi-quantitative concentrations of 0.2–92.5 ng/g and in 21% of samples from surrounding areas (<0.0087–4.06 ng/g). The concentrations of TMBPA exceeded those of the commonly used BPA alternatives, such as BPG and BPZ, suggesting that this emerging BP merits continuous environmental surveillance.

E-waste dismantling facilities were one of the main emission sources of halogenated BPs [30]. TBBPA and other four halogenated BPs (monoClBPA, monoBBPA, DiBBPA, TriBBPA) were 100% detected in e-waste soil. TBBPA and monoBBPA also had detection rates higher than 80% from the surrounding area. The concentrations of TBBPA in e-waste soil in our study (mean, 2250 ng/g) were comparable with those detected in e-waste outdoor dust from Taizhou (mean, 1998 ng/g) [31]. Although the median concentrations of TBBPA were higher than those of its debromination products, including monoBBPA, DiBBPA, and TriBBPA, they exhibited extremely high concentrations in some e-waste samples. For example, the concentration of monoBBPA (17,100 ng/g) collected from a sampling site in the dismantling operation area was approximately three times higher than that of TBBPA (6390 ng/g). This may be due to the fact that debromination is likely to occur during the disposal of e-waste by processes such as heating [10]. However, in the surrounding area, the concentrations of TBBPA in surface soil were consistently higher than those of monoBBPA.

### 3.3. Spatial Distribution of BPs around E-Waste Dismantling Area and Source Implications

**Figure 1:**
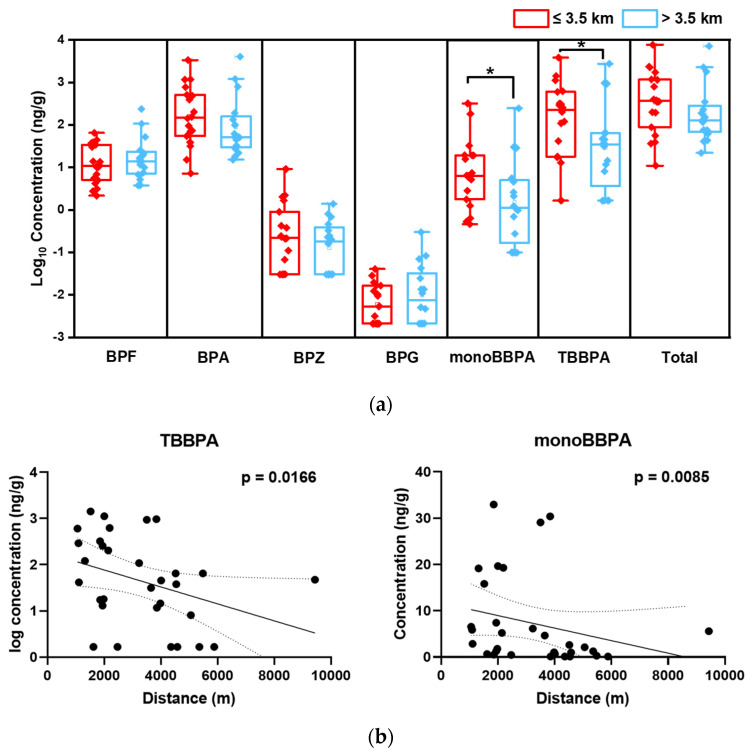
(**a**) Concentrations of BPs in the surface soil from surrounding areas within and beyond the distance of 3.5 km from the e-waste dismantling facilities; * indicates *p* < 0.05; (**b**) the relationship between concentrations of TBBPA and monoBBPA in the surface soil from surrounding areas and the distance from the e-waste dismantling facilities to the sampling sites.

Spatial distributions of BPs with detection frequencies higher than 60% were analyzed to investigate the influences of e-waste dismantling activities on the BPs in the surrounding environment. Samples from the surroundings were divided into two groups according to the distances between the e-waste dismantling facilities: one was ≤3.5 km and the other was >3.5 km. Except for BPF and BPG, all BPs showed lower concentrations at sampling points with distances > 3.5 km (Figure 1a). However, only the results of monoBBPA were significant. Further, correlations between the concentrations of individual BPs and the distances were conducted. Concentrations of TBBPA and monoBBPA show significantly negative correlations with the distances, and the Spearman coefficients (*r_s_*) were −0.363 (*p* = 0.035) and −0.384 (*p* = 0.025), respectively (Figure 1b). Although not significant, the concentrations of BPA and BPZ also decreased as the distance between the sampling sites and e-waste dismantling parks increased. Similar distribution trends with decreasing concentrations and increasing distances have also been observed for halogenated flame retardants in samples from the same e-waste recycling areas [32]. These findings further illustrate the importance of e-waste dismantling activities for the emission of BPs to the environment. Following emissions from e-waste dismantling, these BPs may enter the atmosphere and be deposited in the surrounding area through dry and wet deposition of atmospheric particulate matter. The absence of such trends for some bisphenols may be attributed to the fact that they have more diversified sources in the surrounding areas, such as consumer products.

To further explore the potential sources of BPs in the e-waste dismantling facilities and the surrounding areas, correlations between individual chemicals in soil from two regions were determined. There were significant positive correlations among all identified BPs in e-waste soil except for BPZ, with the correlation coefficient ranging from 0.44 to 0.95 (Figure S7). These findings suggest common applications of a diverse array of BPs in the materials of e-waste. The results of a recent study validate this speculation. The study showed the simultaneous detection of high concentrations of nine BP analogs in e-waste samples [33]. The absence of correlations for BPZ suggests that it has other unique sources that merit further attention. There were also significant correlations between all chemicals including BPZ in the surrounding area samples (Figure S8), suggesting they had similar sources. As the previous results show, the emissions from e-waste dismantling facilities might be the important ones.

### 3.4. Risk Assessment and Health Implications

**Figure 2:**
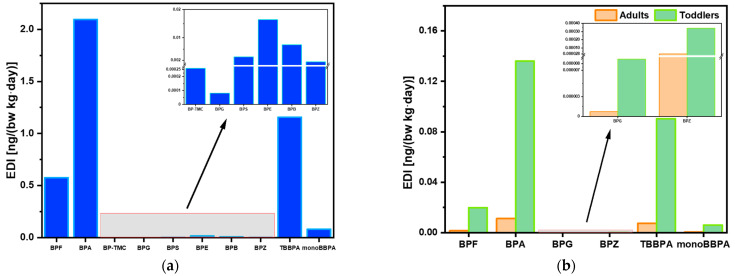
Estimated daily intake (EDI; ng/kg bw/day) of BPs via oral ingestion for (**a**) e-waste dismantling workers, and (**b**) toddlers and adults living in the surrounding areas.

The EDIs of BPs for e-waste dismantling workers and residents (adults and toddlers) via oral ingestions of soil were estimated (Figure 2, Table S5). Occupational workers have the highest exposure, far more than general toddlers and adults living in the surrounding areas. Taking BPA as an example, the median EDI was 2.09 ng/kg bw/day for workers, and it was almost 200 times the EDI of the adult residents (0.01 ng/kg bw/day) and 20 times the EDI of toddler residents (0.14 ng/kg bw/day). BPA and TBBPA contributed the majority of the total EDIs for the three populations. Notably, in the highest exposure scenarios, monoBBPA surpassed BPA and TBBPA as the largest contributor to the total exposure of workers, accounting for approximately 36.7% of total EDIs. However, currently, no studies have indicated the toxicity of monoBBPA, which warrants attention for future research.

In both the average and highest exposure scenarios, the EDIs of BPA were far lower than the temporary TDI (4 μg/kg·bw/day). The HQs of other BPs with available TDIs were also thousands of times lower than one (Table 2). Therefore, no considerable health risks are expected from the exposure to BPs via soil ingestion both for workers and residents. However, the EFSA recently proposed a draft TDI for BPA of 0.2 ng/kg bw/day [34]. Even in the lowest exposure scenarios, the EDI of workers (0.3 ng/kg bw/day) exceeded this draft TDI. The median EDI of toddlers (0.13 ng/kg bw/day) was also close to the new TDI, indicating potential health concerns.

The risk of exposure to BPs estimated in this study may be much lower than the actual situation. Firstly, other exposure sources besides soil ingestion, such as inhalation, dermal absorption, and diet, were found to contribute more to internal exposure to BPs [35]. Secondly, due to the lack of corresponding reference doses for most BPs identified in this study, neglecting their contribution to risk may result in underestimation. Furthermore, the mixed toxic effects of exposure to multiple BPs may differ significantly from those of individual BPs. For example, BPA exposure along with seven other estrogenic chemicals exhibited significant estrogenic activity while each chemical was at a concentration below its effect threshold [36]. Therefore, future research should be more focused on improving the accuracy of risk evaluations and the continued regulation of occupational exposure to BPs.

## 4. Conclusions

In the present study, we performed suspect screening of BPs in e-waste soil, and a total of 14 BPs were identified. Our results demonstrated broad occurrences of BPs in surface soil from the e-waste dismantling facilities and the surrounding areas, suggesting their wide applications. Among all identified BPs, BPA was still the dominant one. The concentrations of TBBPA were also among the highest, and its debromination product, monoBBPA, exhibited extremely high concentrations in some e-waste samples. An emerging alternative of BPA, TMBPA, was identified and showed wide distribution. The spatial distributions of BPs showed that the e-waste dismantling facilities were important sources to the surrounding areas, especially for TBBPA and monoBBPA. The estimation of daily intake via oral ingestion suggests that current contamination scenarios are unlikely to produce considerable exposure risks for e-waste dismantlers and residents. However, the health risk of dismantling workers should not be overlooked given the additional exposure routes and possible mixed exposure effects from co-existing BPs.

## Figures and Tables

**Table 1:** Concentrations and detection frequencies (DF, %) of bisphenol chemicals in surface soil (ng/g) from e-waste dismantling facilities and surrounding areas in South China.

BPs	E-Waste Dismantling Facilities (n = 24)				Surrounding Areas (n = 34)				*p* Value ^3^
	DF	Mean	Median	Range	DF	Mean	Median	Range	
BPA	100	4760	3350	532–16,740	100	460	84.1	7.20–4050	<0.001
BPF	100	1170	920	30.3–4580	100	26.4	12.3	2.17–238	<0.001
BPS	75	11.9	9.86	<0.062–25.0	9	0.75	<0.062	<0.062–14.3	— ^4^
BP-TMC	79	0.83	0.59	<0.058–2.54	21	0.20	<0.058	<0.058–4.79	—
BPG	75	0.84	0.40	<0.0031–3.29	62	0.021	0.005	<0.0031–0.30	<0.001
BPE	67	194	42.5	<0.055–1060	21	0.99	<0.055	<0.055–13.7	—
BPB	100	89.8	11.9	1.05–655	59	1.08	0.17	<0.0045–12.0	—
BPZ	75	20.3	3.75	<0.064–83.4	65	0.66	0.21	<0.064–9.24	0.005
TMBPA ^1^	100	12.8	2.96	0.2–92.5	21	<0.0087	<0.0087	<0.0087–4.06	—
TBBPA	100	2250	1850	268–6390	82	420	55.9	<0.48–3850	<0.001
monoClBPA ^2^	100	136	13.7	0.52–991	12	0.43	<0.48	<0.48–7.77	—
monoBBPA	100	2620	129	12.2–17,140	74	28.7	3.74	<0.48–320	<0.001
DiBBPA ^2^	100	181	6.85	1.11–1020	35	2.96	<0.48	<0.48–49.2	—
TriBBPA ^2^	100	65.6	8.21	0.71–387	38	4.61	<0.48	<0.48–80.4	—

^1^ semi-quantified using a reference standard of BPA; ^2^ semi-quantified using a reference standard of TBBPA; ^3^ comparison between samples from the dismantling facilities and the surrounding area; ^4^ detection rate below 60% without statistical analysis.

**Table 2:** The hazard quotients of BPs via oral ingestion for e-waste dismantling workers and toddlers and adults living in the surrounding areas.

	E-Waste Dismantling Workers			Adults			Toddlers		
	Min	Median	Max	Min	Median	Max	Min	Median	Max
BPF	5.4 × 10^−6^	1.6 × 10^−4^	8.2 × 10^−4^	8.3 × 10^−8^	4.7 × 10^−7^	9.1 × 10^−6^	1.0 × 10^−6^	5.7 × 10^−6^	1.1 × 10^−4^
BPA	8.3 × 10^−5^	5.2 × 10^−4^	2.6 × 10^−3^	2.4 × 10^−7^	2.8 × 10^−6^	1.4 × 10^−4^	2.9 × 10^−6^	3.4 × 10^−5^	1.6 × 10^−3^
TBBPA	1.7 × 10^−7^	1.2 × 10^−6^	4.0 × 10^−6^	2.2 × 10^−10^	7.5 × 10^−9^	5.1 × 10^−7^	2.7 × 10^−9^	9.0 × 10^−8^	6.2 × 10^−6^
BPS	6.0 × 10^−9^	7.4 × 10^−7^	3.6 × 10^−6^						

## Data Availability

The original contributions presented in the study are included in the article/supplementary material, further inquiries can be directed to the corresponding author.

## Supplementary Materials

The following supporting information can be downloaded at https://www.mdpi.com/article/10.3390/toxics12060379/s1: References [37,38] are cited in the Supplementary Material. Table S1: Detailed information on sampling sites; Table S2: Information of identified bisphenol chemicals; Table S3: Parameters used for the estimation of daily intake via dust ingestion; Table S4: Multiple reaction monitoring (MRM) ions of each bisphenol chemical; Table S5: The EDIs of BPs for e-waste dismantling workers and residents (adults and toddlers) via soil ingestion; Figure S1: Distribution of sampling sites for the surface soil samples; Figure S2: Identification of 3,3′,5-tribromobisphenol A in e-waste soil; Figure S3: Identification of monochlorobisphenol A in e-waste soil; Figure S4: Identification of dibromobisphenol A in e-waste soil; Figure S5: Identification of tetramethyl bisphenol A in e-waste soil; Figure S6: Compositions of bisphenol chemicals in surface soil from e-waste dismantling facilities and surrounding areas in South China; Figure S7: Spearman correlations between individual bisphenol chemicals in surface soil from e-waste dismantling facilities; Figure S8: Spearman correlations between individual bisphenol chemicals in surface soil from e-waste dismantling park surrounding areas.

## References

[1] Chen D., Kannan K., Tan H., Zheng Z., Feng Y.-L., Wu Y., Widelka M. (2016). Bisphenol Analogues Other Than BPA: Environmental Occurrence, Human Exposure, and Toxicity—A Review. Environ. Sci. Technol..

[2] Liao C., Kannan K. (2014). A survey of bisphenol A and other bisphenol analogues in foodstuffs from nine cities in China. Food Addit. Contam. A.

[3] Vandenberg L.N., Hauser R., Marcus M., Olea N., Welshons W.V. (2007). Human exposure to bisphenol A (BPA). Reprod. Toxicol..

[4] Hahladakis J.N., Iacovidou E., Gerassimidou S. (2023). An overview of the occurrence, fate, and human risks of the bisphenol—A present in plastic materials, components, and products. Integr. Environ. Assess. Manag..

[5] Bonefeld-Jørgensen E.C., Long M., Hofmeister M.V., Vinggaard A.M. (2007). Endocrine-disrupting potential of bisphenol A, bisphenol A dimethacrylate, 4-n-nonylphenol, and 4-n-octylphenol in vitro: New data and a brief review. Environ. Health Perspect..

[6] Naveira C., Rodrigues N., Santos F.S., Santos L.N., Neves R.A.F. (2021). Acute toxicity of bisphenol a (BPA) to tropical marine and estuarine species from different trophic groups. Environ. Pollut..

[7] Wang X., Nag R., Brunton N.P., Siddique M.A.B., Harrison S.M., Monahan F.J., Cummins E. (2022). Human health risk assessment of bisphenol A (BPA) through meat products. Environ. Res..

[8] Kemikalieinspektionen (2017). Bisfenoler—En Kartläggning Och Analys. https://www.kemi.se/global/rapporter/2017/rapport-5-17-bisfenoler-en-kartlaggning-och-analys.pdf.

[9] Catenza C.J., Farooq A., Shubear N.S., Donkor K.K. (2021). A targeted review on fate, occurrence, risk and health implications of bisphenol analogues. Chemosphere.

[10] Liu J., Ma S., Lin M., Tang J., Yue C., Zhang Z., Yu Y., An T. (2020). New Mixed Bromine/Chlorine Transformation Products of Tetrabromobisphenol A: Synthesis and Identification in Dust Samples from an E-Waste Dismantling Site. Environ. Sci. Technol..

[11] Xu X., Zeng X., Boezen H.M., Huo X. (2015). E-waste environmental contamination and harm to public health in China. Front. Med..

[12] Papaoikonomou K., Emmanouil C., Vasilato V., Diapouli E., Grigoratos T., Zafirakou A., Kungolos A. (2018). PM10 and elemental concentrations in a dismantling plant for waste of electrical and electronic equipment in Greece. Aerosol Air Qual. Res..

[13] Zhang T., Xue J., Gao C.-z., Qiu R.-l., Li Y.-x., Li X., Huang M.-z., Kannan K. (2016). Urinary concentrations of bisphenols and their association with biomarkers of oxidative stress in people living near e-waste recycling facilities in China. Environ. Sci. Technol..

[14] Chakraborty P., Sampath S., Mukhopadhyay M., Selvaraj S., Bharat G.K., Nizzetto L. (2019). Baseline investigation on plasticizers, bisphenol A, polycyclic aromatic hydrocarbons and heavy metals in the surface soil of the informal electronic waste recycling workshops and nearby open dumpsites in Indian metropolitan cities. Environ. Pollut..

[15] Pan Y., Xie R., Wei X., Li A.J., Zeng L. (2024). Bisphenol and analogues in indoor dust from E-waste recycling sites, neighboring residential homes, and urban residential homes: Implications for human exposure. Sci. Total Environ..

[16] Wei D., Yuan K., Ai F., Li M., Zhu N., Wang Y., Zeng K., Yin D., Bu Y., Zhang Z. (2023). Occurrence, spatial distributions, and temporal trends of bisphenol analogues in an E-waste dismantling area: Implications for risk assessment. Sci. Total Environ..

[17] Liu X., Lv Q., Song X., Chen Y., Zhao L., Yan M., Hu B., Chen D. (2023). Screening for bisphenol chemicals: A strategy based on dansyl chloride derivatizatio n coupled with in-source fragmentation by high-resolution mass spectrometry. Anal. Chem..

[18] Liao C., Liu F., Guo Y., Moon H.-B., Nakata H., Wu Q., Kannan K. (2012). Occurrence of eight bisphenol analogues in indoor dust from the United States and several Asian countries: Implications for human exposure. Environ. Sci. Technol..

[19] EFSA Panel on Food Contact Materials, Enzymes, Flavourings and Processing Aids (CEF) (2015). Scientific opinion on the risks to public health related to the presence of bisphenol A (BPA) in foodstuffs. EFSA J..

[20] Barghi M., Shin E.-S., Kim J.-C., Choi S.-D., Chang Y.-S. (2017). Human exposure to HBCD and TBBPA via indoor dust in Korea: Estimation of external exposure and body burden. Sci. Total Environ..

[21] Lyu Z., Harada K.H., Kim S., Fujitani T., Hitomi T., Pan R., Park N., Fujii Y., Kho Y., Choi K. (2023). Temporal trends in bisphenol exposures and associated health risk among Japanese women living in the Kyoto area from 1993 to 2016. Chemosphere.

[22] Zheng J., Yang J., Zhao F., Peng B., Wang Y., Fang M. (2023). CIL-ExPMRM: An Ultrasensitive Chemical Isotope Labeling Assisted Pseudo-MRM Platform to Accelerate Exposomic Suspect Screening. Environ. Sci. Technol..

[23] Qi Y., He J., Xiu F.-R., Yu X., Gao X., Li Y., Lu Y., Song Z. (2019). A convenient chemiluminescence detection for bisphenol A in E-waste dismantling site based on surface charge change of cationic gold nanoparticles. Microchem. J..

[24] Xu Y., Hu A., Li Y., He Y., Xu J., Lu Z. (2021). Determination and occurrence of bisphenol A and thirteen structural analogs in soil. Chemosphere.

[25] Vasiljevic T., Harner T. (2021). Bisphenol A and its analogues in outdoor and indoor air: Properties, sources and global levels. Sci. Total Environ..

[26] Yang Y., Shi Y., Chen D., Chen H., Liu X. (2022). Bisphenol A and its analogues in paired urine and house dust from South China and implications for children’s exposure. Chemosphere.

[27] Qiu W., Zhan H., Hu J., Zhang T., Xu H., Wong M., Xu B., Zheng C. (2019). The occurrence, potential toxicity, and toxicity mechanism of bisphenol S, a substitute of bisphenol A: A critical review of recent progress. Ecotoxicol. Environ. Saf..

[28] Kitamura S., Suzuki T., Sanoh S., Kohta R., Jinno N., Sugihara K., Yoshihara S.i., Fujimoto N., Watanabe H., Ohta S. (2005). Comparative study of the endocrine-disrupting activity of bisphenol A and 19 related compounds. Toxicol. Sci..

[29] Liu Q., Shao W., Weng Z., Zhang X., Ding G., Xu C., Xu J., Jiang Z., Gu A. (2020). In vitro evaluation of the hepatic lipid accumulation of bisphenol analogs: A high-content screening assay. Toxicol. Vitr..

[30] Malkoske T., Tang Y., Xu W., Yu S., Wang H. (2016). A review of the environmental distribution, fate, and control of tetrabromobisphenol A released from sources. Sci. Total Environ..

[31] Wu Y., Li Y., Kang D., Wang J., Zhang Y., Du D., Pan B., Lin Z., Huang C., Dong Q. (2016). Tetrabromobisphenol A and heavy metal exposure via dust ingestion in an e-waste recycling region in Southeast China. Sci. Total Environ..

[32] Ge X., Ma S., Zhang X., Yang Y., Li G., Yu Y. (2020). Halogenated and organophosphorous flame retardants in surface soils from an e-waste dismantling park and its surrounding area: Distributions, sources, and human health risks. Environ. Int..

[33] Runde K., Castro G., Vike-Jonas K., González S.V., Asimakopoulos A.G., Arp H.P.H. (2022). Occurrence and sorption behaviour of bisphenols and benzophenone UV-filters in e-waste plastic and vehicle fluff. J. Hazard. Mater..

[34] Lambré C., Barat Baviera J.M., Bolognesi C., Chesson A., Cocconcelli P.S., Crebelli R., Gott D.M., Grob K., Lampi E., EFSA Panel on Food Contact Materials, Enzymes and Processing Aids (CEP) (2023). Re-evaluation of the risks to public health related to the presence of bisphenol A (BPA) in foodstuffs. EFSA J..

[35] Geens T., Aerts D., Berthot C., Bourguignon J.-P., Goeyens L., Lecomte P., Maghuin-Rogister G., Pironnet A.-M., Pussemier L., Scippo M.-L. (2012). A review of dietary and non-dietary exposure to bisphenol-A. Food Chem. Toxicol..

[36] Silva E., Rajapakse N., Kortenkamp A. (2002). Something from “nothing”—Eight weak estrogenic chemicals combined at concentrations below NOECs produce significant mixture effects. Environ. Sci. Technol..

[37] China Environmental Protection Agency (China EPA) (2013). Exposure Factors Handbook of Chinese Population (Adults).

[38] China Environmental Protection Agency (China EPA) (2013). Exposure Factors Handbook of Chinese Population (0–5 Years).

