# Supplementary material for: Bisphenol Chemicals in Surface Soil from E-Waste Dismantling Facilities and the Surrounding Areas: Spatial Distribution and Health Risk
Source: Toxics. 2024 May 23;12(6):379. doi: 10.3390/toxics12060379 (PMC11209086; doi:10.3390/toxics12060379)
Supplement: Supplementary file 1 [file toxics-12-00379-s001.zip › toxics-3002039-supplementary.pdf]

Bisphenol Chemicals in Surface Soil from E-Waste Dismantling Facilities and the Surrounding Areas: Spatial Distribution and Health Risk

Lei Zhao [1], Fengli Zhou [1], Shuyue Wang [1], Yan Yang [2,3,4], Haojia Chen [2,3,4], Xufang Ma [1] and Xiaotu Liu [1,*]

1 Guangdong Key Laboratory of Environmental Pollution and Health, College of Environment and Climate, Jinan University, Guangzhou 510632, China; lei_zhao1998@163.com (L.Z.); zhoufengli@stu2020.jnu.edu.cn (F.Z.); wangshuyue@stu2022.jnu.edu.cn (S.W.); xufang_ma2002@163.com (X.M.)

2 School of Environmental Science and Engineering, Institute of Environmental Health and Pollution Control, Guangdong University of Technology, Guangzhou 510006, China; yangyan1209@gdut.edu.cn (Y.Y.); chenhaojia_gdut@163.com (H.C.)

3 Synergy Innovation Institute of Guangdong University of Technology, Shantou 515041, China

4 Chemistry and Chemical Engineering Guangdong Laboratory, Shantou 515041, China

* Correspondence: liuxiaotu@jnu.edu.cn

Number of pages: 15

Number of figures: 8

Number of tables: 5

Chemical and reagents

Reference standards of a total of 9 bisphenol chemicals were purchased from AccuStandard (New Haven, CT), including 4,4'-(Propane-2,2-diyl)diphenol (bisphenol A, BPA),, tetrabromobisphenol A (TBBPA), 4,4'-Sulfonyldiphenol (bisphenol S, BPS), bis(4-hydroxyphenyl)methane (bisphenol F, BPF),, 4,4′-ethylidenebisphenol (bisphenol E, BPE), 2,2-bis(4-hydroxyphenyl)butane (bisphenol B, BPB), 2,2-bis(4- hydroxy-3-isopropylphenyl) propane (bisphenol G, BPG), 4,4′-cyclohexylidenebisphenol(bisphenol Z, BPZ), and 1,1-bis(4-hydroxyphenyl)-3,3,5-trimethylcyclohexane (bisphenol TMC, BP-TMC). The standard of 3-monobromobisphenol A (monoBBPA) was purchased from MREDA (Beijing, China). Mass labeled reference standards of BPA-d_6 and BPS-^{13}C purchased from AccuStandard (New Haven, CT), and BPF-d_{10} purchased from Toronto Research Chemicals (North York, Canada), were used as surrogate standards. High-performance liquid chromatography grade solvents were purchased from Fisher Scientific (Hanover Park, IL), and DnsCl used as the derivative reagent was obtained from Apollo Scientific (Cheshire, U.K.).

Table S1. Detailed information on sampling sites.

No.	Longitude	Latitude	Distance to the e-waste dismantling park (m)
		e-waste dismantling facilities	
Park 1	23°30'37.3"N	112°59'54.0"E	—
Park 2	23°19'36.5"N	116°21'29.7"E	—
		surrounding areas	
site-1	23°19'36.5"N	116°21'29.7"E	554
site-2	23°19'34.0"N	116°21'49.8"E	1090
site-3	23°19'33.5"N	116°21'38.5"E	1060
site-4	23°19'38.9"N	116°21'47.6"E	1520
site-5	23°19'42.7"N	116°21'47.4"E	2270
site-6	23°19'40.1"N	116°21'47.4"E	1940
site-7	23°20'00.3"N	116°21'49.6"E	3860
site-8	23°19'25.9"N	116°21'14.1"E	4540
site-9	23°19'28.9"N	116°20'44.8"E	5480
site-10	23°18'54.1"N	116°21'38.3"E	3990
site-11	23°18'28.9"N	116°21'54.5"E	3840
site-12	23°19'07.6"N	116°23'08.6"E	5060
site-13	23°18'24.9"N	116°24'04.6"E	1850
site-14	23°19'08.4"N	116°24'23.4"E	2140
site-15	23°18'11.3"N	116°24'49.0"E	3360
site-16	23°17'35.4"N	116°23'09.5"E	3650
site-17	23°32'34.1"N	116°21'40.6"E	2190
site-18	23°17'39.3"N	116°19'51.5"E	3500
site-19	23°18'57.0"N	116°21'06.1"E	1860
site-20	23°15'30.1"N	116°36'00.1"E	1110
site-21	23°18'20.4"N	116°20'31.1"E	9438
site-22	23°18'54.9"N	116°19'50.2"E	1320
site-23	23°19'53.9"N	116°20'30.4"E	1620
site-24	23°20'26.9"N	116°19'52.7"E	1940
site-25	23°20'28.3"N	116°21'03.5"E	4360
site-26	23°20'18.1"N	116°21'39.6"E	3980
site-27	23°20'13.1"N	116°21'48.0"E	3230
site-28	23°20'21.3"N	116°22'06.1"E	1980
site-29	23°20'35.1"N	116°21'48.8"E	2470
site-30	23°20'28.4"N	116°22'29.8"E	5880
site-31	23°20'40.9"N	116°24'07.1"E	4580
site-32	23°21'26.1"N	116°23'09.3"E	4520
site-33	23°21'27.1"N	116°21'52.3"E	5360
site-34	23°20'43.9"N	116°22'06.8"E	4010

Table S2. Information of identified bisphenol chemicals.

Full name	Abbreviation	CAS	m/z after derivation	Retention time (min)	Log Kow[a]	Log Koa[a]	LOQ (ng/g)[b]	LOQ (ng/g)[c]
Bisphenol A	BPA	1980/5/7	695.2244	18.72	3.32	12.7470	0.009	0.029
Bisphenol F	BPF	620-92-8	667.1931	17.32	2.91	12.5820	0.005	0.017
Bisphenol E	BPE	2081/8/5	681.2088	17.98	3.19	12.7400	0.019	0.062
Bisphenol B	BPB	77-40-7	709.2400	19.48	4.13	13.4320	0.017	0.055
Bisphenol S	BPS	80-09-1	717.1394	15.56	1.65	14.6070	0.005	0.015
Bisphenol Z	BPZ	843-55-0	735.2557	20.21	5.00	14.4130	0.019	0.064
Bisphenol TMC	BP-TMC	129188-99-4	777.3026	21.91	6.29	15.3340	0.017	0.058
Bisphenol G	BPG	127-54-8	779.3183	22.19	6.55	15.3990	0.003	0.010
Monobromobisphenol A	monoBBPA	6073-11-6	773.1349	19.48	4.53	14.3560	0.261	0.870
Tetrabromobisphenol A	TBBPA	79-94-7	1006.8665	21.10	7.20	18.2250	0.144	0.480
Monochlorobisphenol A	monoClBPA	74192-35-1	729.1854	19.40	4.29	13.8470	0.144	0.480
3,3',5-tribromobisphenol A	TriBBPA	6386-73-8	928.9560	20.67	6.31	16.9360	0.144	0.480
2,6-dibromobisphenol A/2,2-dibromobisphenol A	DiBBPA	29426-78-6	851.0454	20.15	5.42	15.6470	0.144	0.480
Tetramethyl bisphenol A	TMBPA	5613-46-7	751.2870	21.46	5.83	15.0850	0.009	0.029

[a] Log Kow and Log Koa values were estimated based on the U.S. Environmental Protection Agency Estimation Program Interface (EPI) Suite Version 4.11.; [b]limits of detection, based on 50 mg of soil, [c]limits of quantification, based on 50 mg of soil.

Table S3. Parameters used for the estimation of daily intake via dust ingestion.

	Dismantling workers	Adults	Toddlers	Reference
Body weight (kg)	60	60	9.9	[37][38]
Exposure fraction (%)[a]	37.5%	16.7%	16.7%	[15]
Dust ingestion rate (mg/day)	50	20	50	[15]

[a]hours spent over a day outdoors

Table S4. Multiple reaction monitoring (MRM) ions of each bisphenol chemical.

Chemicals	Q1	Q3	Frag (V)	CE (V)
BPA	695.2	235.1	275	40
	695.2	171.1	275	48
BPF	667.2	156.1	229	60
	667.2	171.1	229	46
BPE	681.2	156.1	45	60
	681.2	171.1	45	50
BPB	709.2	446.1	25	48
	709.2	171.1	25	52
BPS	717.1	483.1	45	42
	717.1	171.1	45	46
BPZ	735.3	501.2	45	42
	735.3	171.1	45	54
BP-TMC	777.3	543.2	45	44
	777.3	170.1	45	60
BPG	779.3	235.1	25	48
	779.3	171.1	25	52
monoBBPA	774.1	234.7	135	40
	774.1	170.9	135	50
TBBPA	1006.9	710.8	91	25
	1006.9	171.1	91	65
TriBBPA	929.0	694.9	135	35
	929.0	170.9	135	50
DiBBPA	851.0	617.0	135	45
	851.0	553.0	135	40
monoClBPA	729.2	431.2	135	45
	729.2	171.1	135	40
TMBPA	751.3	453.3	135	45
	751.3	171.1	135	40
BPS-$^{13}C_{12}$	729.2	156.1	45	60
	729.2	171.1	45	52
BPA-d6	701.3	156.1	45	60
	701.3	171.1	45	48
BPF-d10	677.3	156.1	275	60
	677.3	171.1	275	48

Table S5. The EDIs of BPs for e-waste dismantling workers and residents (adults and toddlers) via soil ingestion.

	e-waste dismantling workers			adults			toddlers		
	Min	Median	Max	Min	Median	Max	Min	Median	Max
BPF	1.89E-02	5.75E-01	2.86E+00	2.89E-04	1.64E-03	3.17E-02	2.67E-03	1.52E-02	2.93E-01
BPA	3.32E-01	2.09E+00	1.05E+01	9.60E-04	1.12E-02	5.40E-01	8.87E-03	1.04E-01	4.99E+00
BPG	1.33E-06	8.09E-05	2.05E-03	2.83E-07	7.18E-07	4.02E-05	2.61E-06	6.62E-06	3.71E-04
BPZ	1.90E-05	1.42E-03	5.21E-02	4.05E-06	2.83E-05	1.23E-03	3.74E-05	2.61E-04	1.14E-02
TBBPA	1.68E-01	1.16E+00	3.99E+00	2.21E-04	7.46E-03	5.14E-01	2.04E-03	6.89E-02	4.74E+00
monoBBPA	7.64E-03	8.04E-02	1.07E+01	1.33E-05	4.98E-04	4.27E-02	1.23E-04	4.60E-03	3.94E-01
BP-TMC	1.38E-05	2.59E-04	1.59E-03						
BPS	2.65E-05	3.26E-03	1.56E-02						
BPE	1.58E-04	1.64E-02	6.64E-01						
BPB	6.56E-04	7.46E-03	4.09E-01						

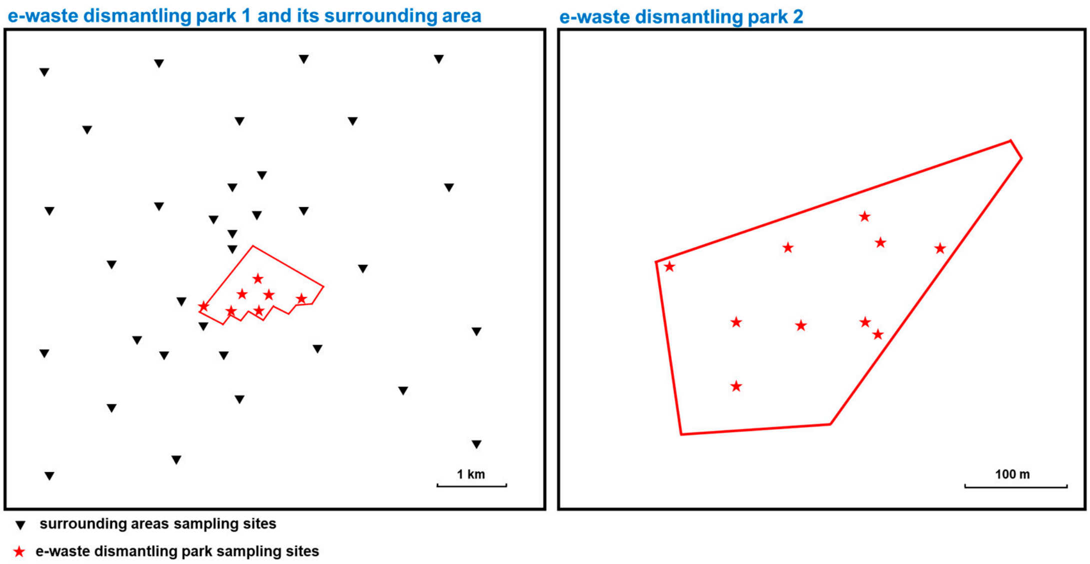

Figure S1. Distribution of sampling sites for the surface soil samples.

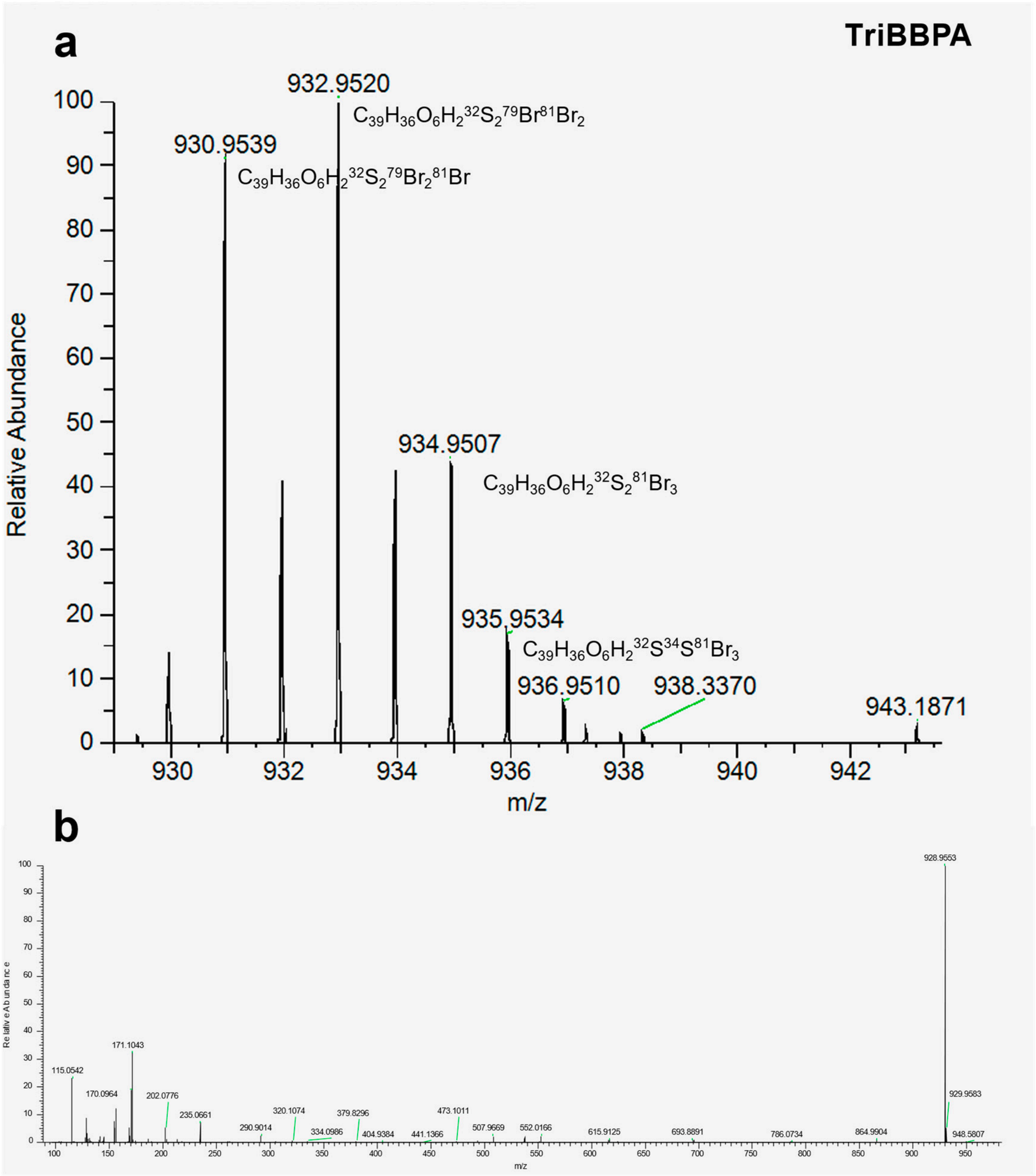

Figure S2. Identification of 3,3',5-tribromobisphenol A in e-waste soil. (a) zoom-in full-scan mass spectrum. (b) DDA MS/MS spectra of *m/z* 928.9558.

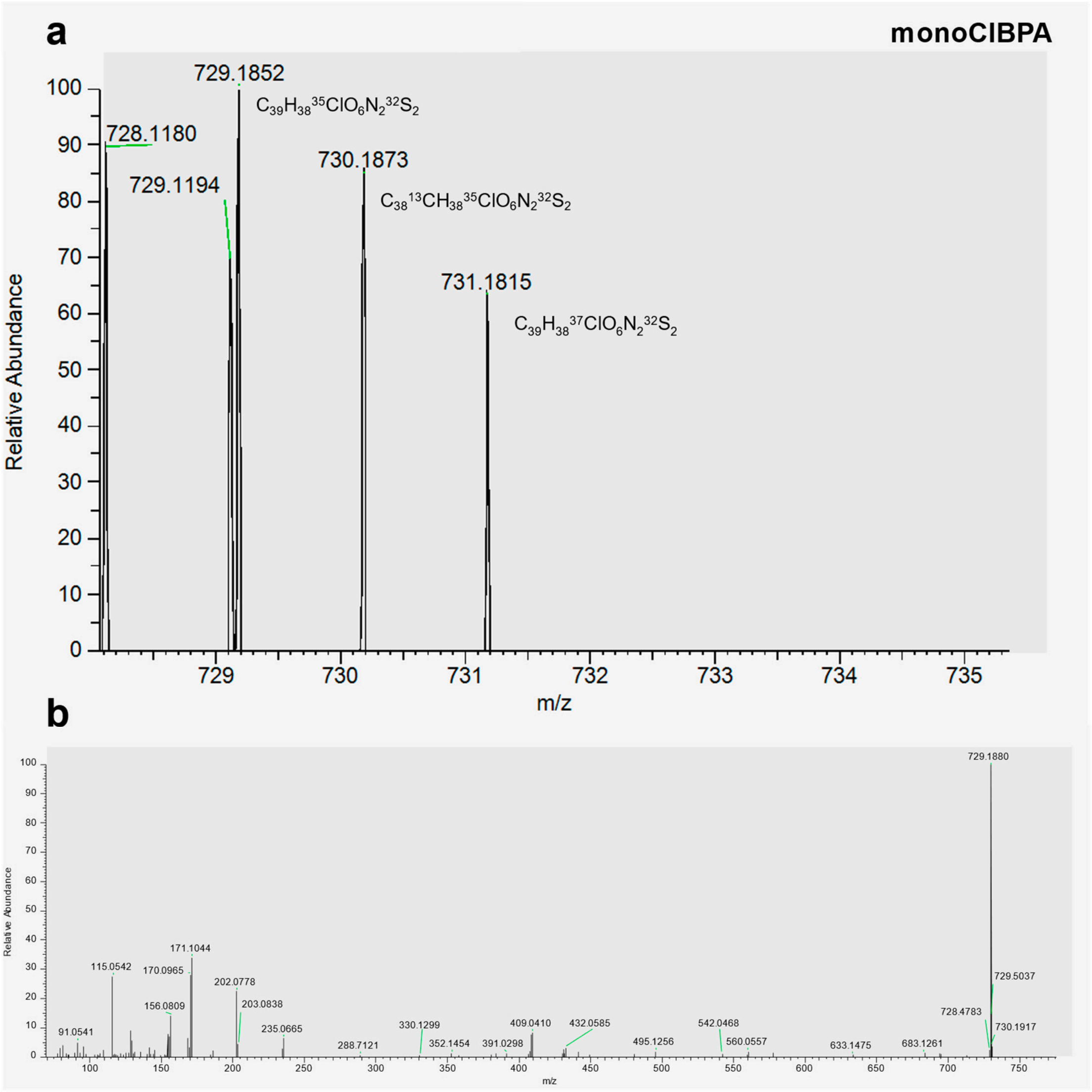

Figure S3. Identification of monochlorobisphenol A in e-waste soil. (a) zoom-in full-scan mass spectrum. (b) DDA MS/MS spectra of *m/z* 729.1880.

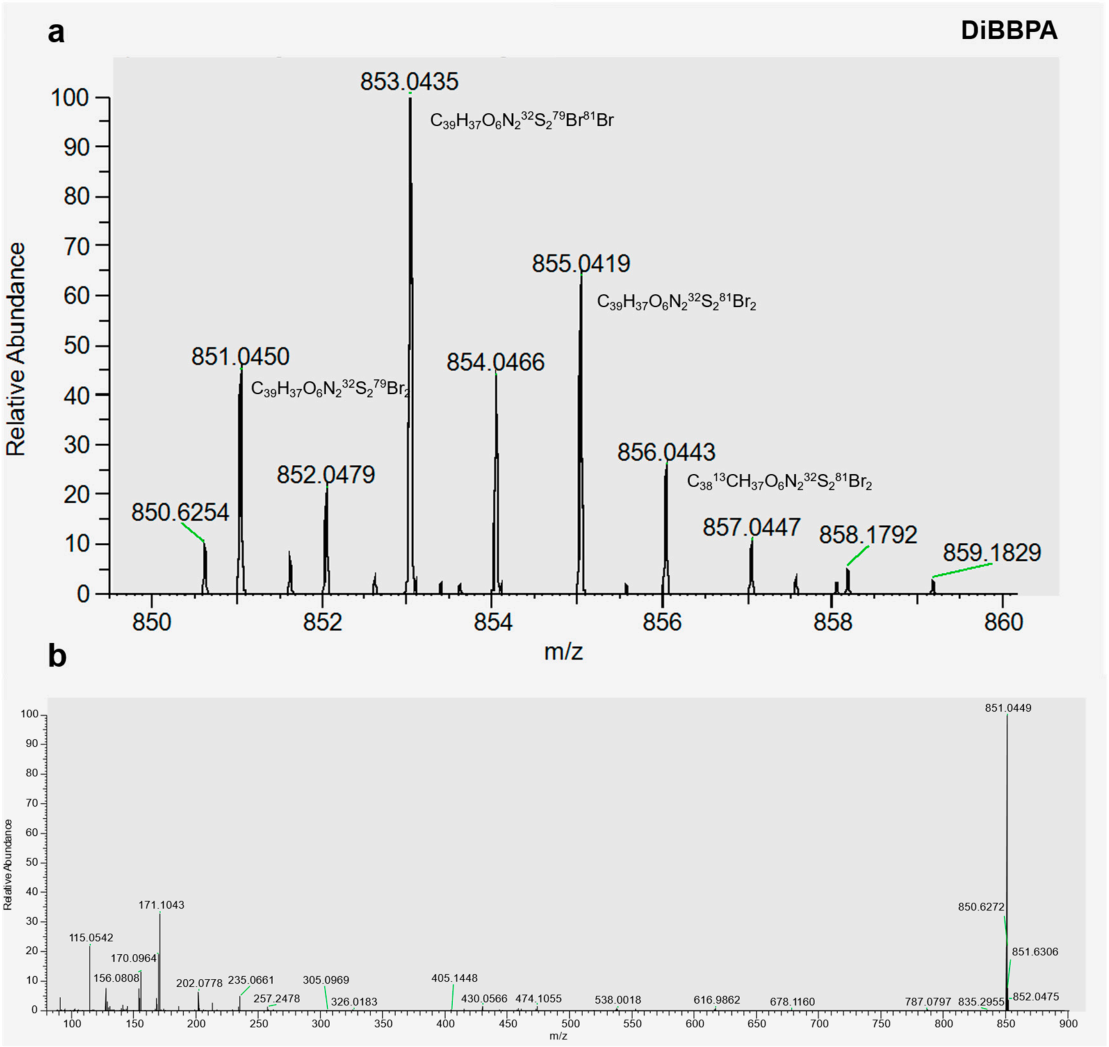

Figure S4. Identification of dibromobisphenol A in e-waste soil. (a) zoom-in full-scan mass spectrum. (b) DDA MS/MS spectra of *m/z* 851.0449.

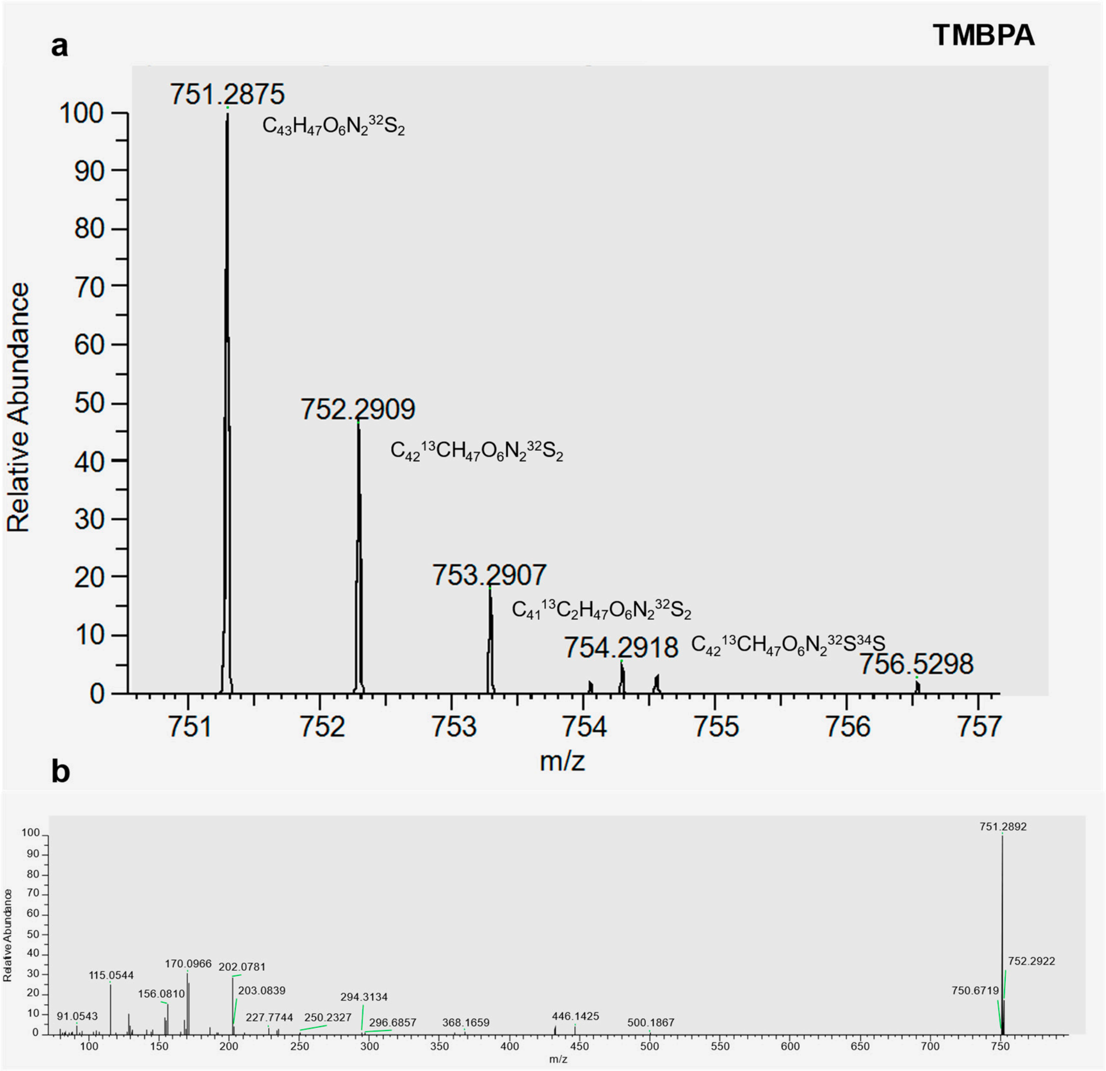

Figure S5. Identification of tetramethyl bisphenol A in e-waste soil. (a) zoom-in full-scan mass spectrum. (b) DDA MS/MS spectra of *m/z* 751.2870.

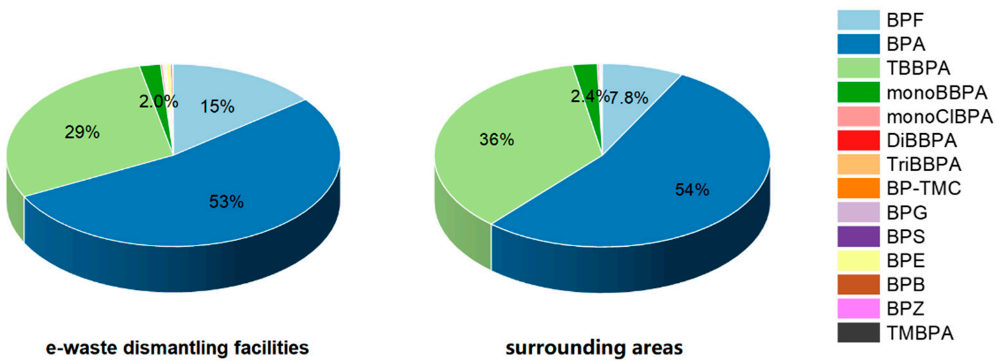

Figure S6. Compositions of bisphenol chemicals in surface soil from e-waste dismantling facilities and surrounding areas in South China.

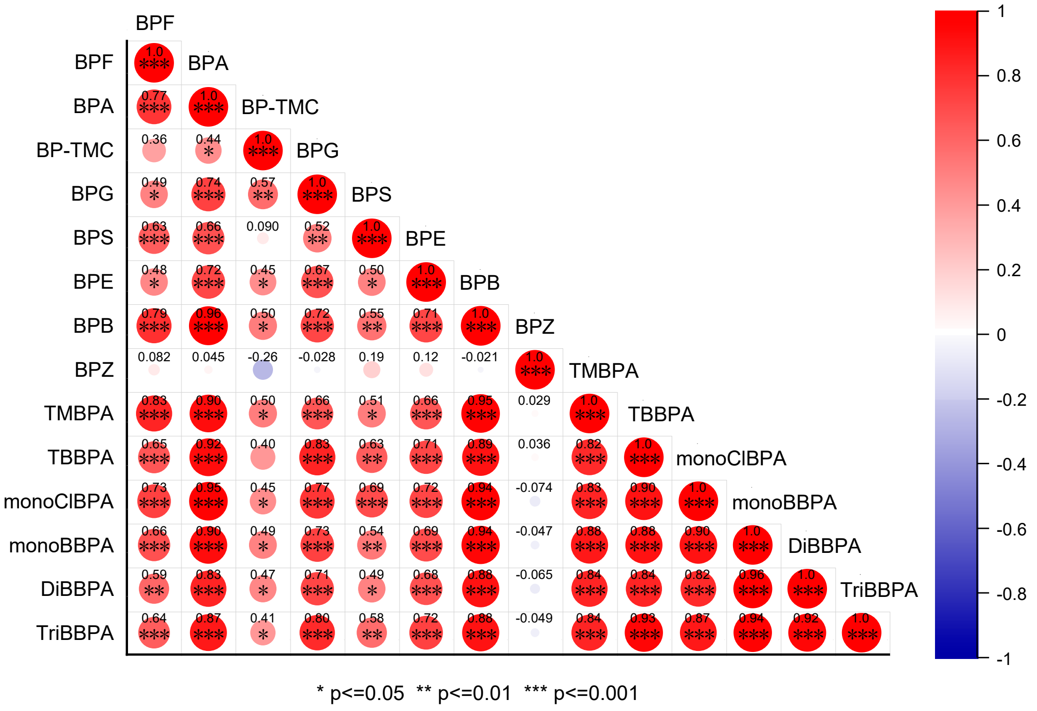

Figure S7. Spearman correlations between individual bisphenol chemicals in surface soil from e-waste dismantling facilities.

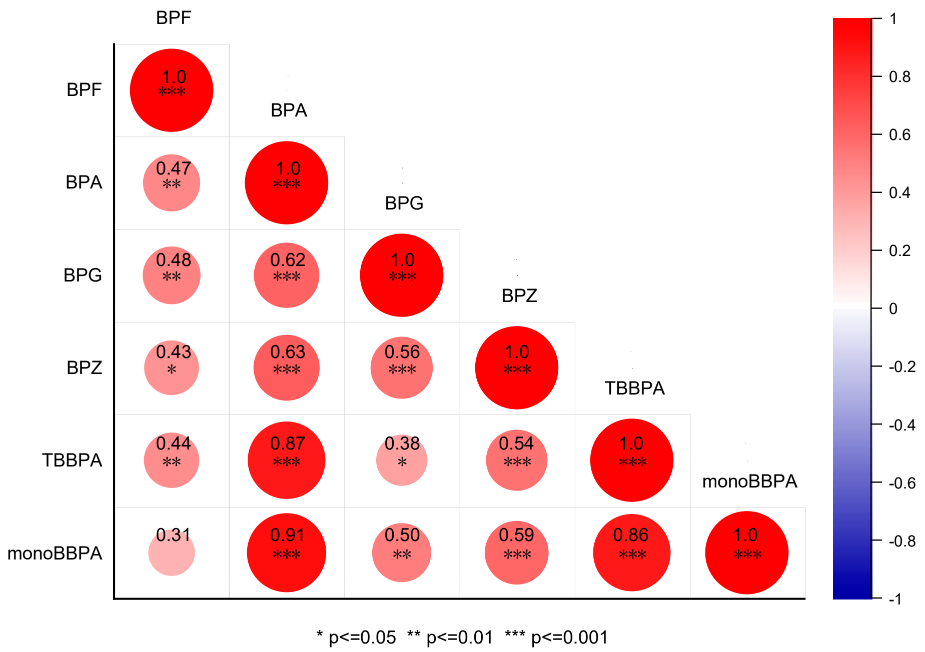

Figure S8. Spearman correlations between individual bisphenol chemicals in surface soil from e-waste dismantling park surrounding areas.
